# Surgical and Visual Outcome for Recurrent Retinal Detachment Surgery

**DOI:** 10.1155/2014/810609

**Published:** 2014-08-11

**Authors:** Constantin Pournaras, Chrysanthi Tsika, Catherine Brozou, Miltiadis K. Tsilimbaris

**Affiliations:** ^1^University Eye Clinic of Geneva, 1211 Geneva, Switzerland; ^2^Department of Ophthalmology, University of Geneva, 1211 Geneva, Switzerland; ^3^Memorial Rothschild Eye Research Unit, La Colline Ophthalmology Center, rue De La Roseraie 75A, 1205 Geneva, Switzerland; ^4^University Eye Clinic of Heraklion, 71500 Heraklion, Greece; ^5^Department of Ophthalmology, University of Larissa, 41110 Larissa, Greece

## Abstract

*Purpose*. To evaluate the anatomical and functional outcome of repeated surgeries for recurrent retinal detachment. *Methods*. We retrospectively reviewed 70 cases with refractory retinal detachment of various etiologies that required multiple operations. Anatomical success (attached retina) or failure (totally/partially-detached retina) was assessed biomicroscopically. The BCVA was used for the evaluation of the functional outcome, at presentation and at the end of follow-up. Various pre-, intra-, and postoperative factors were associated with anatomical success or failure as well as with final functionality. *Results*. The mean number of surgeries was 4 (range: 2 to 10). The anatomical success rate was 80% (56 attached cases, 14 detached cases). 29% of the attached cases had a BCVA better than 20/40 (Snellen chart). The number of operations doesn't seem to affect significantly the final visual acuity. The PVR was found to affect both the anatomical and functional outcome (*P* = 0.014 & *P* = 0.002, respectively). *Conclusions*. In the present study, it is suggested that multiple operations for refractory retinal detachment may result in successful anatomic results, with a fare functional outcome at the same time. Eventually, we verified that the existence of PVR worsens the prognosis.

## 1. Introduction

The advances of vitreoretinal microsurgical techniques of the recent years have facilitated multiple consecutive interventions in a single eye, in cases where the pathology persists. Recurrent retinal detachment (RRD), once a difficult to manage outcome, today represents an indication for repeated surgery. However, the recurrence of a retinal detachment (RD) still represents a factor that is considered to influence unfavorably the final surgical outcome. Several studies have reported the results of specific patients' subgroups that required repeated retinal detachment surgery. However, the overall influence of repeated surgeries is unclear, since only few studies have reported the functional and anatomical results of patients with retinal detachment of various types that required multiple operations. In this paper, we study retrospectively the functional and anatomical results of a series of patients that required repeated operations for the management of retinal detachments related to a variety of pathologies, in the Retina Service of the University Hospital of Geneva.

## 2. Methods

This is a retrospective observational case series in which we reviewed the records of retinal detachment cases and included 70 patients that have been operated on for recurrent retinal detachment in the Retina Service of the Ophthalmology Clinic, University of Geneva, Switzerland. Patients' age ranged from 17 to 83 years (mean 60 years, SD = 13), 56 were males and 14 were females. All patients were operated on by two experienced posterior segment surgeons and had a postoperative follow-up of at least 6 months after the last intervention. All postoperative follow-ups were made at the University Eye Clinic of Geneva.

Data collected from the patient records included patient age and gender; etiology of retinal detachment; best corrected visual acuity (BCVA) before the first intervention; intraoperative techniques and maneuvers used in each patient's consecutive operations such as utilization of scleral buckling (SB), vitrectomy, encircling band (EB), retinectomy, endolaser, cryopexy, gas, and silicone oil (sil-oil); intraoperative vitreous entrapment at the sclerotomy sites; presence of an epiretinal membrane (ERM); presence of proliferative vitreoretinopathy (PVR); discovery of new or missed breaks; postoperative complications; number of operations for each patient; anatomical status of the retina at the end of the follow-up; BCVA at the end of the follow-up; intraocular pressure (IOP) at the end of the follow-up time; and total duration of the follow-up period.

Preoperative and postoperative visual acuity was measured with the Snellen chart. For statistical analysis, visual acuity measurements were converted to logMAR values using appropriate calculations [1, 2].

Patients were divided into 4 groups based on the etiology of their primary retinal detachment as shown in Table 1. Patients having a giant tear detachment or detachments due to high myopia were reported as group 1 (*n* = 14; 13 males, 1 female; mean age 60 years). Patients having a pseudophakic detachment were reported as group 2 (*n* = 31 patients; 25 males and 6 females; mean age 46 years). Patients having a posttraumatic retinal detachment were reported as group 3 (*n* = 7; 6 males and 1 female; mean age 65 years). Finally, phakic patients with primary RD were reported as group 4 (*n* = 18; 12 males, 6 females; mean age 59 years). Anatomical success was considered the total attachment of the retina at the end of the follow-up time. Totally or partially detached retina was considered as failure.

The present research has followed the Tenets of the Declaration of Helsinki.

### 2.1. Statistical Analysis

Patients were divided into two groups based on anatomical success at the end of the follow-up time as defined above: group of attached patients and group of detached patients. The BCVA (in logMAR) at presentation and the end of follow-up was compared with the Wilcoxon Signed Ranks Test for two related samples (as the distribution of the variables was not normal).

Comparison of variables between the two anatomical groups (attached-detached) was assessed with the nonparametric Mann-Whitney *U* Test.

The effect of preoperative variables, the intraoperative maneuvers, and postoperative complications on the anatomical outcome was tested, after appropriate transformation of the variables, with the Chi-square and Fisher's exact test when applicable. After justifying the significant associations, a multivariate analysis was assessed using logistic regression for the factors having significant impact on the outcome.

Further analysis was assessed for the anatomically successful group (i.e., group with attached retina). In this subgroup, the factors that may allow for a final BCVA greater than 20/40 (0.3logMAR) were investigated. Similarly, for these associations, univariate (Chi-square and Fisher's exact test) and multivariate (logistic regression) analysis was attempted.

For all the above, the PASW Statistics 17.0 was used (©2009 SPSS Inc., Chicago, Illinois, USA).

## 3. Results

The postoperative follow-up ranged from 6 to 95 months. The mean number of reoperations was 4, ranging from 2 to 10.

### 3.1. Anatomic Results

Among the 70 cases investigated, 56 (80%) ended up with attached and 14 (20%) with detached retina. The two anatomical groups were compared for the parameters shown in Table 2. Statistical significance was found for the existence of PVR and the performance of retinectomy. When a multivariate analysis was done, using the variables that were found statistically significant in the univariate analysis, only the existence of PVR seemed to have some effect on the anatomical outcome. This effect, however, marginally missed reaching statistical significance (*P* = 0.056).

### 3.2. Functional Results

The functional outcome, in terms of BCVA, was examined in the group with successful anatomical outcome (attached retina). The average visual acuity at the last follow-up in the group of attached patients was 0.9logMAR (20/160 Snellen) compared to a vision of 1.4logMAR (20/500 Snellen) at their initial presentation. This represents an improvement of 0.5logMAR units and it was statistically significant (*P* = 0.039).

The cut-off of 20/40 Snellen (0.3logMAR) was used to evaluate the factors that may predispose for moderate to high final visual acuity. Same parameters as in previous comparison were used and are shown in Table 3. Among the fifty-six cases with attached retina, sixteen (29%) ended with a BCVA better than 0.3logMAR. The number of operations wasn't found to have any association with the improved visual performance. The existence of PVR, the use of silicon oil, and the use of scleral buckling were found to significantly affect the final BCVA. However, when entering the multivariate logistic regression model, only the existence of PVR was proven significant for the final functional outcome.

## 4. Discussion

This study investigated a series of patients with retinal detachment that required multiple operations, independent of etiology. The aim of the study was to evaluate the potential role of repeated surgery in patients with recurrent disease, in terms of anatomic and functional success. All the eyes had recurrent retinal detachment and underwent further surgery (from two to ten operations). This study attempts to clarify if the increased number of surgeries affects the final outcome and to identify factors that may influence this outcome.

In this study, 80% of the patients ended up with attached retina. All our patients had one failed RD operation before entering the study. We were able to show that our group, although it underwent multiple operations, ended-up with relatively good anatomical result. Our results are difficult to compare with the literature, because references to eyes that have received multiple operations for RD are mainly indirect. The majority of retinal detachment treatment publications refer either on first operation or on limited number of reoperations for patients with RD of specific etiology or they are focused on specific techniques (such as retinectomy or silicon-oil tamponade and removal) [3–6, 14]. Anatomic success rates in the literature vary from 53 to 81%, in specific patient groups that were treated for recurrent retinal detachment. Only when cases treated successfully with a single operation are included, the percentage of success overcomes 90% [7].

The overall analysis in this study pointed out that the number of operations does not have an unfavorable effect on the final outcome of repeated retinal detachment surgeries. No statistical significance was found when correlating the number of surgeries neither with the anatomical outcome nor with the functional outcome; the BCVA of patients that underwent multiple interventions was not found to be significantly less than those with two operations. Using the univariate correlation, retinectomy and PVR were identified as significant factors for the anatomic outcome; PVR and the use of silicon oil and scleral buckling were found to be significant factors for the functional outcome. The risk factor that consistently emerged as significant when applying multivariate analysis was the existence of proliferative vitreoretinopathy for both the anatomical and functional result.

In univariate analysis, a negative association was found between the anatomic success (attached retina) and both the existence of PVR (*P* = 0.014) and the performance of retinectomy (*P* = 0.039); among patients that ended up detached, 86% had PVR and 79% underwent retinectomy. Proliferative vitreoretinopathy (PVR) represents the major cause of failure of retinal detachment surgery [3, 8–13]. Expectedly enough, the existence of PVR proved to be the major reason for unfavorable outcome in our study also. In the total of 70 patients, 54% had PVR grade C2 or higher. Among the detached cases, 86% had PVR grade C2 or higher, whereas 46% of the attached cases had PVR grade C2 or higher. Furthermore, we found that retinectomy was a significant factor for anatomical failure. Correlations in literature about retinectomy are contradictory, depending again on the selection of patients. la Heij et al. [6] found that the size of retinectomy matters, correlating retinectomy greater than 180 degrees with anatomic failure. Other investigators [7, 14], on the other hand, showed that retinectomy is important for complicated cases with PVR. These reports together with the findings of our study may indicate that retinectomy may be an indispensible surgical maneuver for difficult cases but the need for its utilization indicates complexity of the case and is associated with worse surgical prognosis.

The number of reoperations was not found to be a significant factor neither for the anatomical nor for the functional outcome in this study. This is in contrast to other studies that consider multiple interventions as significant factor for unsuccessful outcome. Large series with patients that have had multiple operations for retinal detachment [5, 14, 15] found a significant influence of the number of surgeries on the recurrence of the detachment. However, the same authors could not identify the number of operations as a risk factor for poor visual performance (VA less than 6/24). Further research with larger number of cases may be necessary in order to clarify the effect of repeated retinal detachment surgery in anatomical and functional outcomes.

In general, the functional outcome after repeated retinal detachment surgery is considered poor [16] even in cases that have successful anatomical result. In the present study, we evaluated the functional outcome of the anatomically successful cases choosing a relatively high cut-off value of final visual acuity compared to the majority of relevant studies (0.30logMAR or 20/40 in the Snellen chart) [6, 7, 14, 16, 17]. Even with this high cut-off value we were able to show that even after multiple recurrences and operations, the retina is capable of retaining quite good functionality if it is finally maintained attached. Among our patients with attached retina, 29% had a BCVA better than 0.30logMAR (20/40 Snellen chart) at the end of the follow-up. This is considerably greater than the values reported in other studies with similar criteria (5%-[7]; 13%-[4]). Studies with higher percentages of “good visual acuity” (12%–59%) have all used lower cut-offs, ranging from 0.6logMAR (12%-[14]) and 0.7logMAR (47%-[18]) to 1.00logMAR (51%-[12]; 59%-[17]).

As for the factors that affect the visual outcome in attached cases, the univariate analysis, in addition to the unfavorable effect of PVR, revealed the use of scleral buckling as favorable and the use of silicone oil as unfavorable factor for a final BCVA <0.3logMAR. The silicone-oil tamponade is often reported to result in decreased BCVA [6, 12, 14, 15, 17, 19]. In our study, only the 31% (*P* = 0.022) among patients with anatomic success that underwent silicone oil tamponade had good visual acuity (<0.3logMAR). The usual practice of reserving silicon oil tamponade for the more difficult and complicated cases may be an explanation of the less favorable functional results of these cases. On the other hand, scleral buckling showed a positive influence on the final functional outcome; among the attached patients, those that had undergone scleral buckling achieved a good visual acuity in a proportion of 56% (*P* = 0.043). It is possible that cases that were handled with scleral buckle represent cases that were initially judged as less severe and this may explain the relatively better functional result.

In conclusion, in this study, we were able to show that repeated operations in cases of refractory retinal detachment can result in a high percentage of anatomical success. Among the attached eyes, a considerable percentage retained a good visual acuity. The analysis of our data showed that repeated surgery does not have an unfavorable influence in anatomical and functional outcome. Although PVR seems to be the main factor of anatomical failure and poor functional results, our data support that, in cases of unsuccessful retinal detachment operations, surgeons need to continue with as many additional interventions as technically feasible.

## Figures and Tables

**Table 1:** Demographics of the study group.

*N*	Age mean (SD)	Gender	Eye
Total (70)	61 (13)	M: 54 F: 16	OD: 36 OS: 34

Pseudophakics (31)	65 (11)	M: 26 F: 05	OD: 16 OS: 15

Phakics (39)			
HM/GT^a^ (14)	59 (12)	M: 12 F: 02	OD: 05 OS: 09
Trauma (7)	46 (15)	M: 07 F: 00	OD: 06 OS: 01
PRRD^b^ (18)	60 (12)	M: 11 F: 07	OD: 10 OS: 08

^a^HM: high myopia, GT: giant tears.

^b^PRRD: primary rhegmatogenous retinal detachment, other than trauma, high myopia, or giant tears.

**Table 2:** Pre- and intraoperative factors affecting the final anatomic outcome.

Pre- and intraoperative risk factors	Attached (total *N* = 56)	Detached (total *N* = 14)	Chi-square	Multivariate logistic regression
	*n* (%)	*n* (%)	(*P* value)	(*P* value)
*N* of operations				
2	16 (29)	1 (0.07)	NS^a^	
>2	40 (71)	13 (99.93)		
PVR				
<C2	30 (54)	2 (14)		
≥C2	26 (46)	12 (86)	**0.014** ∗	NS (0.056)
Category				
(1) Pseudophakic	24 (43)	07 (50)		
(2) HM/GT^b^	13 (23)	01 (7)		
(3) Traumatic	06 (11)	01 (7)		
(4) PRRD^c^	13 (23)	05 (36)	NS	
Macular pathology	5 (0.09)	0 (0)	NS	
Retinectomy	26 (46)	11 (79)	**0.039** ∗	NS
Silicon oil	31 (0.55)	12 (83)	NS (0.063)	
Gas	49 (88)	11 (79)	NS	
Cryopexy	38 (68)	9 (64)	NS	
Endolaser	33 (60)	12 (83)	NS (0.071)	
Vitreous entrapment	6 (0.11)	0 (0)	NS	
Secondary breaks	21 (38)	5 (36)	NS	
Scleral paracentesis	6 (0.11)	2 (0.14)	NS	
Encircling band	29 (52)	8 (57)	NS	
Scleral buckling	20 (36)	4 (29)	NS	

^a^NS: nonsignificant result.

^b^HM: high myopia, GT: giant tears.

^c^PRRD: primary rhegmatogenous retinal detachment in phacik patients, other than trauma, high myopia, or giant tears.

∗Significant difference (*P* < 0.05).

**Table 3:** Pre- and intraoperative factors affecting the BCVA in the anatomically successful cases.

Pre- and intraoperative risk factors	Number of attached subjects *N* = 56	BCVA ≥0.3log⁡MAR *N* = 40	BCVA ≤0.3log⁡MAR *N* = 16	Chi-square	Multivariate logistic regression
	*n* (%)	*n* (%)	*n* (%)	(*P* value)	(*P* value)
*N* of operations				NS^a^	
2	16 (29)	9 (23)	7 (44)		
>2	40 (71)	31 (77)	9 (56)		
PVR				**0.002** ∗	**0.024** ∗
<C2	30 (54)	16 (40)	14 (88)		
≥C2	26 (46)	24 (60)	2 (12)		
Category				NS	
(1) Pseudophakic	24 (43)	18 (45)	6 (37)		
(2) HM/GT^b^	13 (23)	9 (22.5)	4 (25)		
(3) Traumatic	06 (11)	3 (7.5)	3 (19)		
(4) PRRD^c^	13 (23)	10 (25)	3 (19)		
Macular pathology	5 (9)	3 (8)	2 (13)	NS	
Retinectomy	26 (46)	22 (55)	4 (25)	NS	
Silicon oil	31 (55)	26 (65)	5 (31)	**0.022** ∗	0.472
Gas	49 (88)	35 (88)	14 (88)	NS	
Cryo	38 (69)	25 (63)	13 (81)	NS	
Laser	33 (59)	24 (60)	9 (56)	NS	
Secondary breaks	21 (38)	14 (35)	7 (44)	NS	
Vitreous entrapment	6 (11)	5 (13)	1 (6)	NS	
Scleral paracentesis	6 (11)	5 (13)	1 (6)	NS	
Encircling band	29 (52)	20 (50)	9 (56)	NS	
Scleral buckling	20 (36)	11 (28)	9 (56)	**0.043** ∗	0.135

^a^NS: nonsignificant result.

^b^HM: high myopia, GT: giant tears.

^c^PRRD: primary rhegmatogenous retinal detachment in phacik patients, other than trauma, high myopia, or giant tears.

∗Significant difference (*P* < 0.05).
